# Morphological, olfactory, and vocal development in big brown bats

**DOI:** 10.1242/bio.201410181

**Published:** 2014-12-12

**Authors:** Heather W. Mayberry, Paul A. Faure

**Affiliations:** Department of Psychology, Neuroscience & Behavior, McMaster University, Hamilton, ON L8S 4K1, Canada

**Keywords:** Chiroptera, *Eptesicus fuscus*, Frequency modulated (FM), Harmonics, Olfactory discrimination, Vespertilionidae

## Abstract

Using a within subjects design, we documented morphological, bioacoustical and behavioral developmental changes in big brown bats. *Eptesicus fuscus* pups are born naked and blind but assume an adult-like appearance by post-natal day (PND) 45 and flight by PND 30. Adult females use spatial memory, acoustic and olfactory cues to reunite with offspring, but it is unclear if pups can recognize maternal scents. We tested the olfactory discrimination abilities of young *E. fuscus* pups and found they exhibited no odor preferences. Pups also emit distinct vocalizations called isolation calls (i-calls) that facilitate mother-offspring reunions, but how pups shift their vocalizations from i-calls to downward frequency modulated (FM) sweeps used in echolocation remains unclear. Between PND 0–9, pups emitted mainly long duration, tonal i-calls rich in harmonics, but after they switched to short duration, downward FM sweeps with fewer harmonics. Call maximum frequency and repetition rate showed minor changes across development. Signal duration, bandwidth, and number of harmonics decreased, whereas the maximum, minimum and bandwidth of the fundamental, and peak spectral frequency all increased. We recorded vocalizations during prolonged maternal separation and found that isolated pups called longer and at a faster rate, presumably to signal for maternal assistance. To assess how PND 13 pups alter their signals during interactions with humans we compared spontaneous and provoked vocalizations and found that provoked calls were spectrally and temporally more similar to those of younger bats suggesting that pups in distress emit signals that sound like younger bats to promote maternal assistance.

## INTRODUCTION

Most bats are insectivorous and roost in large colonies. Due to their highly social and gregarious nature, bats rely on acoustic communication—both self-communication (echolocation) and between conspecifics—for survival and reproduction. Bats are capable of powered flight, hence acoustic communication can occur over some distance. Owing to their nocturnal lifestyle, acoustic communication in bats has undergone sophisticated evolution and is more prominent than visual communication (Nelson, 1964; Gould, 1971; Matsumura, 1979). Bats use vocal signals for indicating distress, interacting with conspecifics, orientation, object avoidance and prey detection hence sound production is particularly important for survival (Fenton, 1985). Bats control many aspects of their echolocation calls such as signal duration, rate and direction of frequency modulation (FM), rate and magnitude of amplitude modulation (AM), signal bandwidth, harmonic structure, and both the number and timing of calls emitted (Gunderson, 1976; Fenton et al., 1987; Fenton et al., 2011).

Female big brown bats (*Eptesicus fuscus*) give birth to one (western populations) or two (eastern populations) young per year (Christian, 1956; Barbour and Davis, 1969; Hood et al., 2002; Kurta and Baker, 1990). During development, pups spontaneously emit distinct i-calls that facilitate maternal localization and retrieval (Gould, 1971; Moss, 1988). When the mother bat leaves the roost at night to forage her relatively immobile pups are typically left behind. Returning mothers are then faced with the problem of locating and identifying their own offspring. In the wild, adult female bats of most species normally do not nurse unrelated pups even though infants will eagerly nurse from any convenient lactating mother (Davis et al., 1968; Turner et al., 1972; Brown, 1976; Porter, 1979; Brown et al., 1983; Thomson et al., 1985; Gustin and McCracken, 1987; but see Kleiman, 1969; Watkins and Shump, 1981; McCracken, 1984; Wilkinson, 1992; de Fanis and Jones, 1996).

Selective nursing necessitates individual recognition between parent and offspring (for a review, see Kunz and Hood, 2000). Previous research has shown that mother-offspring recognition and reunions are a multi-sensory experience, relying on olfactory (e.g. scent marking) and acoustic cues as well as spatial memory (Brown, 1976; Watkins and Shump, 1981; Gustin and McCracken, 1987; McCracken, 1993). Pups emit i-calls with distinct vocal signatures (Rasmuson and Barclay, 1992; Scherrer and Wilkinson, 1993; Knörnschild and von Helversen, 2008). Mother bats use acoustic cues to identify their own offspring, and pups can discriminate calls of their own mother from those of another mother bat (Turner et al., 1972; Brown, 1976; Brown et al., 1983; Gelfand and McCracken, 1986; Balcombe, 1990; Balcombe and McCracken, 1992; Thomson et al., 1985). Moreover, adult males and lactating females of some species can discriminate between their own muzzle odor and that from another male or lactating female conspecific (Gustin and McCracken, 1987). More recently there is evidence that bats have detectable colony-specific odor signatures (de Fanis and Jones, 1995a; Bloss et al., 2002).

Pups emit i-calls until a certain point in their development, after which their vocalizations change to mainly downward FM sweeps (chirps) used for echolocation. Precursor vocalizations to FM sweeps have been observed in infancy while pups are still emitting i-calls (Gould, 1971; Moss, 1988). Precursor vocalizations are signals with similar characteristics to both i-calls and adult echolocation calls, but are less sophisticated in their acoustic structure. It is unclear how pup vocalizations shift from being predominately i-calls to broadband, downward FM sweeps, and whether pup calls are precursors to adult echolocation or social vocalizations (de Fanis and Jones, 1995b). Moreover, the principle acoustic features that mother's use to recognize their offspring are still unknown (Bohn et al., 2007).

Previous studies on bat vocal development have used pups of unknown or estimated age, focused on a short time period of development, or used a small sample of pups and presented the findings as a between-subjects comparison (e.g. Davis et al., 1968; Davis, 1969; Matsumura, 1979; Matsumura, 1981; Habersetzer and Marimuthu, 1986; Moss et al., 1997; Monroy et al., 2011; Scherrer and Wilkinson, 1993; Sterbing, 2002; Van Parijs and Corkeron, 2002; Vater et al., 2003; Zhang et al., 2005; Bohn et al., 2007; Liu et al., 2007). In this study, we examined the ontogeny of vocal development in a large sample of captive-born big brown bat pups studied from the day of birth (PND 0) to PND 25. We then used a within-subjects design to document detailed temporal, spectral and behavioral changes during development. We also ran a series of experiments to see if *E. fuscus* pups were capable of recognizing and discriminating odors of their mother *versus* another reproductive (i.e. lactating) or non-reproductive adult female, either from the same or different colony as the mother. Finally, we conducted two additional experiments—the first to determine if isolated pups alter their vocalizations during prolonged periods of maternal separation and the second to compare the acoustic parameters of spontaneous *versus* provoked vocalizations—to test the hypothesis that distressed pups can alter their signals to mimic the vocal characteristics of younger bats to promote immediate assistance and/or retrieval by their mother.

## RESULTS

### Morphology and Behavior

#### Pup Growth

**Fig. 1.**
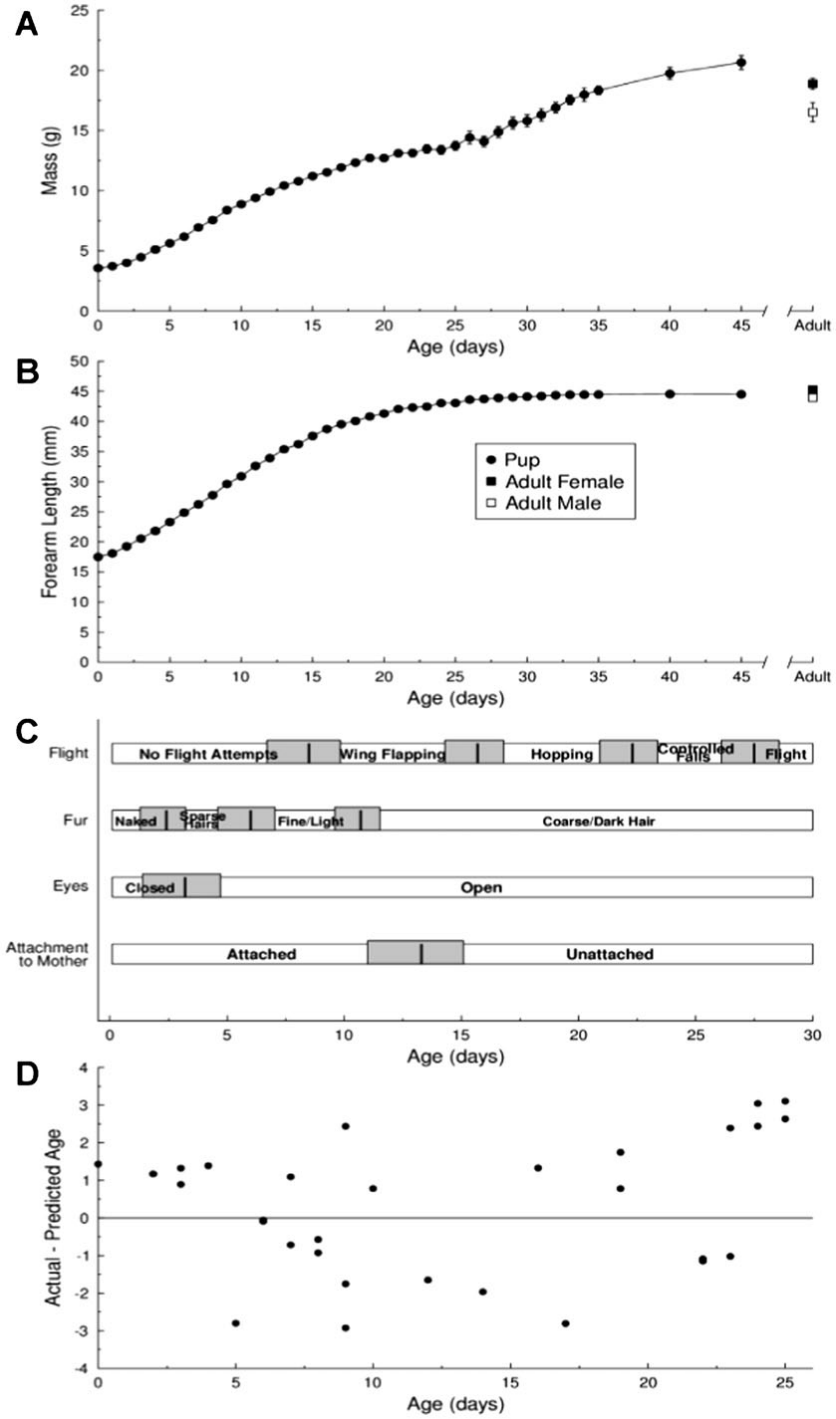
Pup growth and milestones of development. (A) Mean ± SE change in pup mass (n = 15–28 pups) and (B) forearm length (n = 15–75 pups) with age. *Closed* and *open squares* are the mean ± SE female (n = 128) and male (n = 40), respectively, mass and forearm lengths measured from wild-caught adult bats. (C) Timeline illustrating important milestones of pup development. Horizontal bars represent different aspects of morphological and behavioral changes: whether pup was found attached to the mother, the opening of the eyes, absence/presence of fur and type of hair, and flying ability. *Black vertical lines* represent the mean age in days after birth when different milestones were achieved; *grey shading* represent ± 1 standard deviation (SD). (D) Difference between the actual and estimated age of bat pups determined with the growth equations developed for pups ranging from PND 0 to PND 25. Estimated age calculated as the average age of the mass and forearm length growth equations (see text).

Captive-born pup mass increased nearly linearly throughout development (Fig. 1A). There was no difference in mass between male (3.45±0.14 g, n = 11) and female (3.65±0.12 g, n = 15) pups at birth (t = 1.13, d.f. = 21, p = 0.269) or by PND 45 (20.1±1.46 g (n = 5); 21.1±0.59 g (n = 10); t = 0.65, d.f. = 5, p = 0.546). The average increase in mass between PND 0–25 was 0.41 g/day, but was lower when calculated from PND 0–45 (0.38 g/day). The variance in pup mass also increased with age (Fig. 1A). By PND 45 the average mass of captive-born male and female pups was slightly higher than the average mass of wild-caught adult male and female *E. fuscus* (wild adult male mass: 16.5±0.79, n = 40, t = 2.27, d.f. = 10, p = 0.047; wild adult female mass: 18.9±0.47; n = 128; t = 2.99, d.f. = 23, p = 0.006). Wild-caught adult females were also heavier than wild-caught adult males (t = 2.57, d.f. = 68, p = 0.012; Fig. 1A); however the difference in mass was almost surely because the females were pregnant.

The forearm length of captive-born pups also increased linearly early in development but then eventually plateaued. The average growth of forearm length from PND 0–25 was 1.05 mm/day but slowed to 0.60 mm/day when considering PND 0–45. There was also a slight increase in the variance of the forearm length data with age (Fig. 1B). There was no difference in forearm length at birth between captive-born male (17.44±0.18, n = 33) and female (17.55±0.17, n = 34) pups (t = 0.43, d.f. = 64, p = 0.670), or by PND 45 (45.01±0.60 mm, n = 5; 44.43±0.48 mm, n = 10; t = −0.76, d.f. = 9, p = 0.464). There was also no difference in forearm length between male and female PND 45 pups and wild-caught adult males (43.98±0.32 mm, n = 40; t = 0.98, d.f. = 8, p = 0.356) and females (45.25±0.17 mm, n = 128; t = −1.63, d.f. = 12, p = 0.128). The average forearm length of wild-caught adult males was smaller than that of wild-caught adult females (t = 3.48, d.f. = 63, p = 0.001; Fig. 1B).

#### Developmental Milestones

Big brown bat pups follow relatively stable morphological and behavioral developmental trajectories (Fig. 1C). Because newborn pups are altricial they are very dependent on their mother for nutrition, warmth and protection and are consistently found attached to and nursing from her until PND 13/14. As pups mature, their eyes open, they grow fur, and eventually develop the motor skills necessary for flight. Newborn pups are born with their eyes closed but the eyes usually open on PND 2/3. Pups are born hairless and remain naked until PND 3/4. After PND 4, they begin to grow sparse hairs on their lower back and stomach, and by PND 7/8 these hairs cover the entire body. The fur is fine and light colored until PND 8/9, after which it becomes dark and coarse. Adult-like fur is typically seen by PND 10/11. Behaviorally, pups were not observed to make flight attempts until PND 7/8, after which they began to flap their wings when hung by their feet and encouraged to fly. By PND 13 pups began wing hopping but were unable to perform controlled falls (descents) or flight attempts until PND 21. Most pups did not achieve true powered flight until PND 27/28.

#### Growth Equations and Age Estimation

We developed growth equations from a sub-sample of the mass and forearm length data that we collected from healthy male and female pups from PND 0−15 so that we could directly compare our results to those obtained by Burnett and Kunz (Burnett and Kunz, 1982) who developed growth equations from the same age range. Two ages were estimated for each pup—one with the mass equation (age = −1.78667 _*_ (3.0916 − mass)) and another with the forearm length equation (age = −0.705517 _*_ (16.614 − forearm length)). We averaged these to give a final estimated age for each pup. The average absolute difference between a pup's actual age and its predicted age for bats between PND 0–15 using these equations was 1.22±0.18 days (n = 30, range = days).

We also developed mass and forearm length growth equations using a different sub-sample of the data that extended the age range to PND 25 (Fig. 1D).
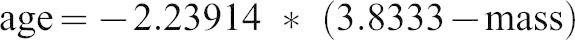
(1)
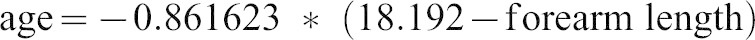
(2)With these new equations, the average absolute difference between a pup's actual age and its predicted age between PND 0–25 was 1.58±0.16 (n = 30, range = days).

### Olfactory Discrimination Trials

Most pups spent an equal amount of time near both odor test areas within the runway. Table 1 displays the proportion of time pups spent in proximity to each scent in our test pairings. In total, we analyzed the results from 6 scent pairings testing 79 pups across three age groups (PNDs 0–7, 8–14, and 15–20). We found no evidence that *E. fuscus* pups displayed olfactory preferences or that pups preferred the odor of their mother *versus* any other odor.

**Table 1.**
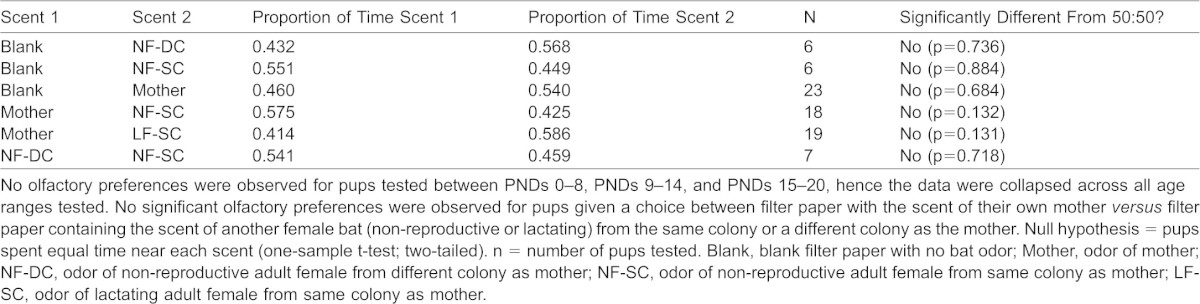
Summary of results of 2-AFC olfactory discrimination testing in *E. fuscus* pups

### Vocal Recordings

#### Call Types

**Fig. 2.**
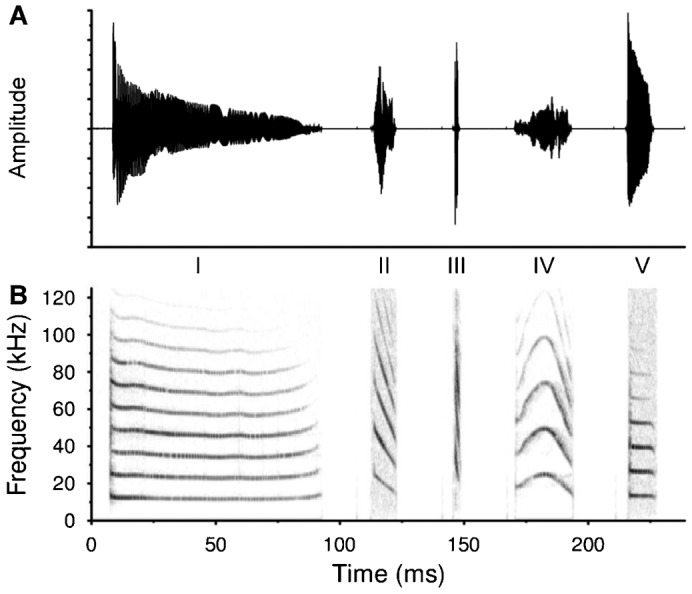
Exemplar call types emitted by developing big brown bat pups. Oscillogram (A) and spectrogram (B) views of five call types. From left to right: Type I (i-call), Type II (intermediate transition call), Type III (echolocation call), Type IV (social-like call), and Type V (short duration tonal call with small FM). Although the time base in both panels is accurate, the intercall interval is not because the signals were copied and pasted to construct the figure.

Fig. 2 shows a composite oscillogram (*top*) and spectrogram (*bottom*) illustrating the five different call types recorded from developing *E. fuscus* pups. Table 2 shows the qualitative and quantitative descriptions of each call type.

**Table 2.**
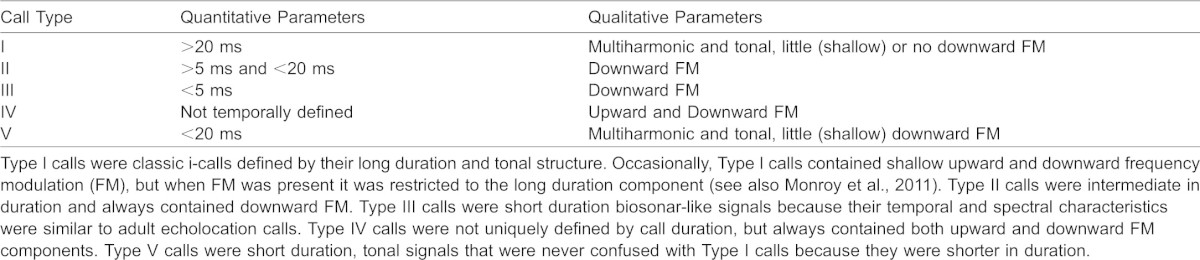
Quantitative and qualitative parameters used to classify the calls of *E. fuscu*s pups

**Fig. 3.**
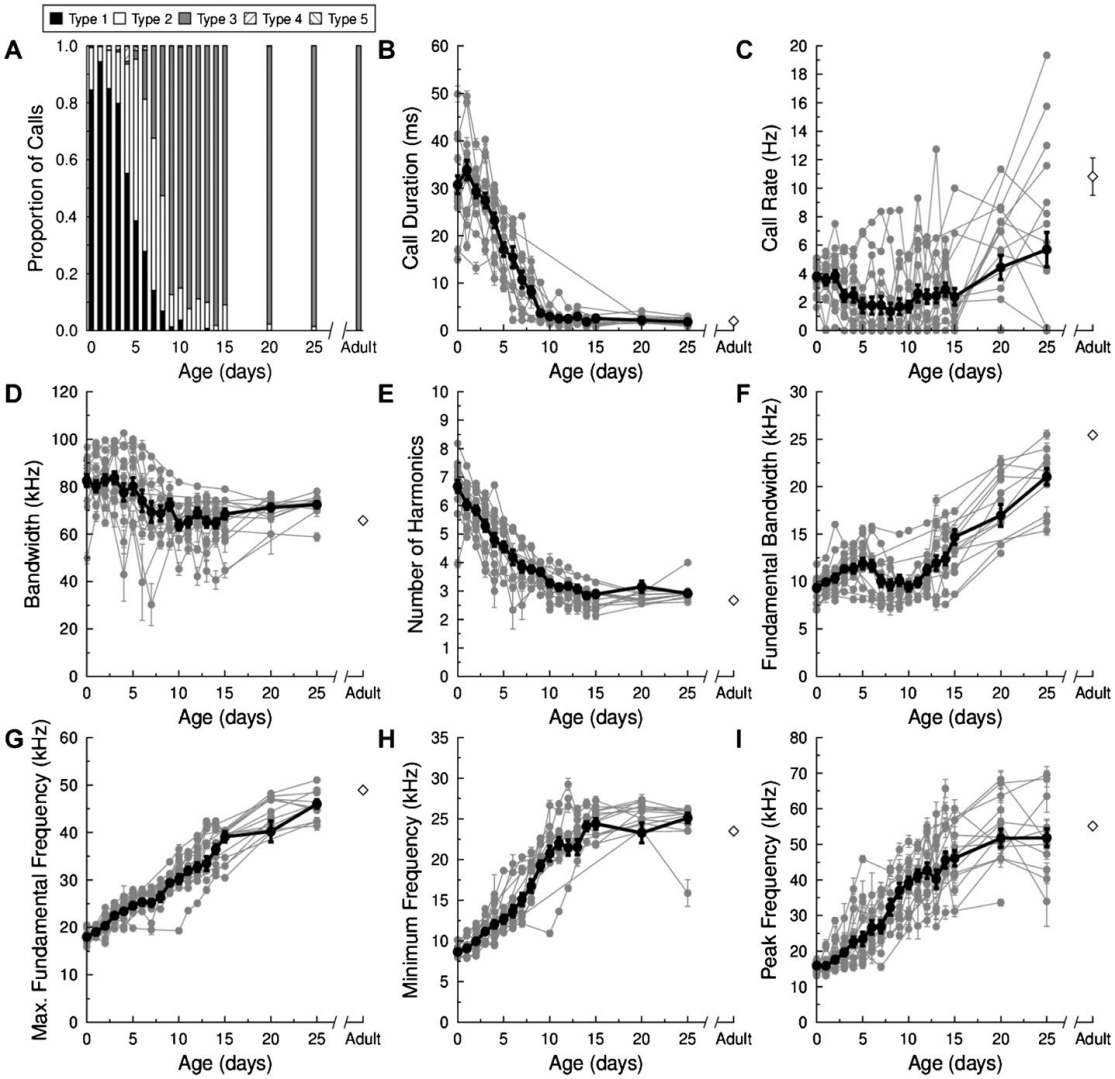
Change in call types and their acoustic parameters by *E. fuscus* pups during development. (A) Distribution of calls type proportions emitted by pups from birth (PND 0) through PND 25 of development (n = 16–22 pups) compared to wild-caught adults (n = 9). Change in (B) call duration, (C) call repetition rate, (D) total signal bandwidth, (E), number of harmonic elements, (F) bandwidth of the fundamental acoustic element, (G) maximum fundamental frequency, (H) minimum frequency, and (I) peak spectral frequency as a function of age during development. In panels B–I, the *grey lines* are data from 15 individual pups and the *black lines* are the mean ± SE values across development; the *open diamonds* are the mean ± SE values measured from 9 wild-caught adults.

The use of the different call types changed dramatically throughout development (Fig. 3). In the first days of life (mean age = 2.2 days) approximately 80 to 90% of all pup vocalizations were Type I isolation calls, but i-call production was almost nonexistent by PND 15 (Fig. 3A). Type II calls were also relatively common early in development, with peak production occurring between PND 5–8 (mean age = 6.1 days) and declining rapidly after PND 9/10. Type II calls were mainly emitted by pups transitioning between Type I and Type III calls (Fig. 3A). Type III calls were not part of the vocal repertoire until PND 5, but were common in bats aged two weeks and older (mean age = 14.1 days). Type III calls were similar to biosonar calls emitted by adult *E. fuscus*. Indeed, almost all vocalizations emitted after PND 10 were classified as Type III calls. Type IV and Type V calls were relatively rare and were mainly seen early in development. The average ages of pups emitting Type IV and Type V calls were 4.0 and 4.1 days, respectively. Despite possessing a rich repertoire of social vocalizations (Gadziola et al., 2012), all vocalizations recorded from adult big brown bats were classified as Type III calls (Fig. 2, Fig. 3A).

#### Pup Vocal Parameters

Fig. 3 also details how the acoustic parameters of *E. fuscus* pup vocalizations changed during development. The panels in Fig. 3B–H show both individual curves and the population averages for the data from call Types I, II, and III; data from call Types IV and V were not included because these vocalizations were relatively infrequent. Pups produced long duration (30 ms) i-calls immediately after birth, with call durations decreasing sharply by PND 10 and remaining short until adulthood (Fig. 3B). Although there was considerable individual variation, the average spontaneous rate of calling remained more or less constant throughout development (Fig. 3C). After birth, young pups emitted i-calls at a rate of approximately 4 Hz, decreasing to ca. 1.5–2 Hz by PND 10, and then increasing to 5–6 Hz by PND 25. The spontaneous rate of calling of adult bats was approximately 10.8 Hz (Fig. 3C). Despite wide changes in signal duration with age, total signal bandwidth decreased only slightly from birth until adulthood (Fig. 3D). Call bandwidths in the youngest pups were ca. 80–85 kHz, decreasing to 70–75 kHz by PND 25. Bandwidths of adult vocalizations were approximately 65 kHz. The number of harmonic elements decreased dramatically during the transition from Type I to Type III calls (Fig. 3E). Young pups emitted i-calls containing an average of 7 harmonic elements, decreasing to ca. 3 elements by PND 13/14. The biosonar-like calls of adult bats typically contained 2–3 harmonics (Fig. 2, Fig. 3E). The remaining panels in Fig. 3F–I detail spectral changes in call Types I, II, and III. Although total signal bandwidth decreased slightly with age (Fig. 3D), the bandwidth of the fundamental acoustic element increased from 10 to 20 kHz between PND 0–25 (Fig. 3F). Adult bats emitted calls with a mean fundamental bandwidth of 25 kHz (Fig. 3F). Call maximum frequency remained relatively constant throughout development (data not shown). Very young pups emitted calls with maximum frequencies between 90–95 kHz; older pups and adults emitted calls with equivalent maximum frequencies. The maximum fundamental frequency, minimum fundamental (and call) frequency, and peak spectral frequency all increased nearly linearly with age, eventually reaching adult values (Fig. 3F–H).

The data in Fig. 3B–I describe how the acoustic parameters of call Types I, II and III changed with age. Here we describe differences between call Types I, II, and III with respect to average spectral bandwidth, number of harmonics, and peak spectral frequency (data not shown). Type I calls were larger in bandwidth than Type II (t = 25.81, d.f. = 5384, p = 1.3E−138) or Type II calls (t = 66.8, d.f. = 5701, p = 0), and Type II calls were larger in bandwidth than Type III calls (t = 31.73, d.f. = 6363, p = 2.9E−205). Type I calls contained more harmonic elements than Type II (t = 62.2, d.f. = 6179, p = 0) or Type III calls (t = 183.19, d.f. = 10006, p = 0), and Type II calls had more harmonic elements than Type III calls (t = 73.27, d.f. = 4933, p = 0). Type I calls had a lower peak spectral frequency than Type II (t = −32.63, d.f. = 4143, p = 4.5E−208) and Type III calls (t = −83.92, d.f. = 10746, p = 0), and Type II calls had a lower peak spectral frequency than Type III calls (t = −2.22, d.f. = 3532, p = 0.027).

### Prolonged Isolation Recordings

**Fig. 4.**
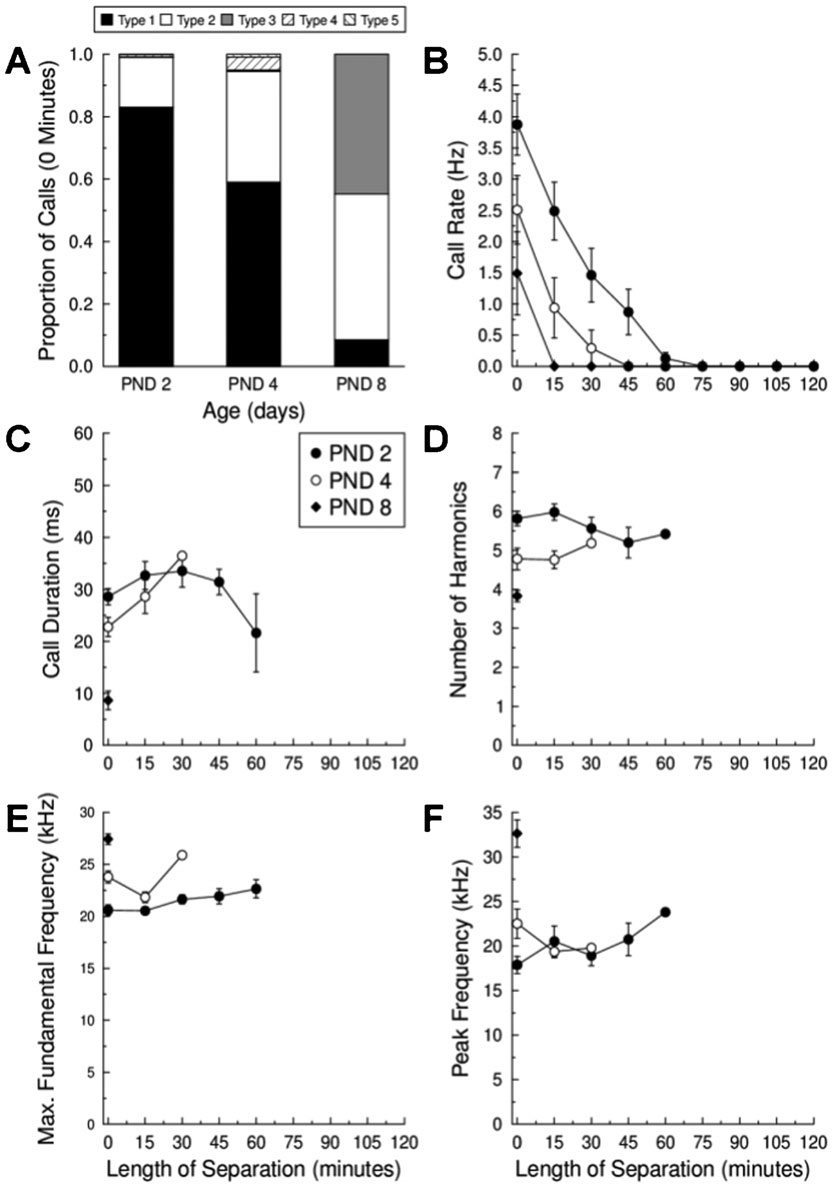
Change in call types and their acoustic parameters by *E. fuscus* pups during prolonged maternal separation. (A) Distribution of call type proportions emitted by PND 2, PND 4, and PND 8 pups immediately after separation from their mother (data from 15, 14, and 7 pups were averaged to produce the proportions on PND 2, PND 4 and PND 8, respectively). Mean ± SE change in (B) call repetition rate, (C) call duration, (D) number of harmonic elements, (E) maximum fundamental frequency, and (F) peak spectral frequency as a function of maternal separation time for PND 2 (*closed circles*), PND 4 (*open circles*), and PND 8 (*closed diamonds*), respectively. Note: PND 8 pups ceased calling after 0 min of maternal separation.

Fig. 4 shows the results of an experiment designed to test the hypothesis that young *E. fuscus* pups would be more likely to vocalize than older pups during prolonged separation due to their greater need for maternal assistance. Call type distribution of isolated PND 2, 4 and 8 pups after 0 min of maternal separation was different (χ^2^ = 1252.58, d.f. = 6, p<0.001). PND 2 pups emitted mainly Type 1 i-calls immediately after separation, whereas PND 4 pups emitted a higher proportion of Type II calls and PND 8 pups emitted Type II and Type III calls more or less equally with few Type I calls (Fig. 4A).

The rate of calling changed dramatically as a function of age and maternal separation time (Fig. 4B). PND 2 pups had higher average calling rates immediately after separation than PND 4 and PND 8 pups, and PND 4 pups called at a higher rate than PND 8 pups (F = 8.86, d.f. = 28, p = 0.001). There was also a difference in the time after maternal separation at which pups ceased to emit calls (i.e. call rate = 0 Hz). PND 2 pups continued to call for much longer after separation than PND 4 (t = 10.68, d.f. = 6, p = 0.00004) or PND 8 pups (t = 4.73, d.f. = 6, p = 0.003). There was also a trend for PND 8 pups to cease calling more quickly than PND 4 pups during prolonged maternal separation; however, the difference was not quite significant (t = 2.33 , d.f. = 6, p = 0.059).

Immediately after maternal separation, PND 2 pups emitted longer duration calls than PND 4 and PND 8 pups, and PND 4 pups also emitted longer duration calls than PND 8 pups (Fig. 4C; F = 32.62, d.f. = 12, p = 1.407E−5). These results were consistent with our previous developmental data (Fig. 3B). Call durations of PND 2 pups emitted 30 minutes after separation were not different from call durations emitted at separation (t = −1.96, d.f. = 9, p = 0.082). Call durations emitted by PND 4 pups at 0 and 15 minutes of separation were also not different (t = −1.28, d.f. = 3, p = 0.289). PND 8 pups called immediately after being separated from their mother but did not emit any calls thereafter, hence it was not possible to compare any acoustic parameters at later time points.

Except for spectral differences resulting from testing pups of different ages (e.g. Fig. 3D–I), prolonged maternal separation did not cause overt changes in pup vocalizations throughout the separation time (Fig. 4D–F). There was no change in the number of harmonics (Fig. 4D) emitted by PND 2 pups between 0 and 45 min of maternal separation (t = 1.62, d.f. = 5, p = 0.166), or by PND 4 pups between 0 and 15 min of maternal separation (t = 1.03, d.f. = 3, p = 0.379). There was no difference in the maximum fundamental frequency (Fig. 4E) emitted by PND 2 pups between 0 and 45 minutes of separation (t = −0.24, d.f. = 6, p = 0.815), or by PND 4 pups between 0 and 15 minutes of separation (t = 2.69, d.f. = 3, p = 0.075). And there was no difference in the peak spectral frequency (Fig. 4F) emitted by PND 2 pups after 0 and 45 minutes of separation (t = −1.41, d.f. = 6, p = 0.208), or by PND 4 pups after 0 and 15 minutes of separation (t = −2.07, d.f. = 3, p = 0.130). Again, no PND 8 pups emitted calls after they were isolated from their mother so it was not possible to test for acoustic differences as a function of separation time.

### Spontaneous vs. Provoked Recordings

**Fig. 5.**
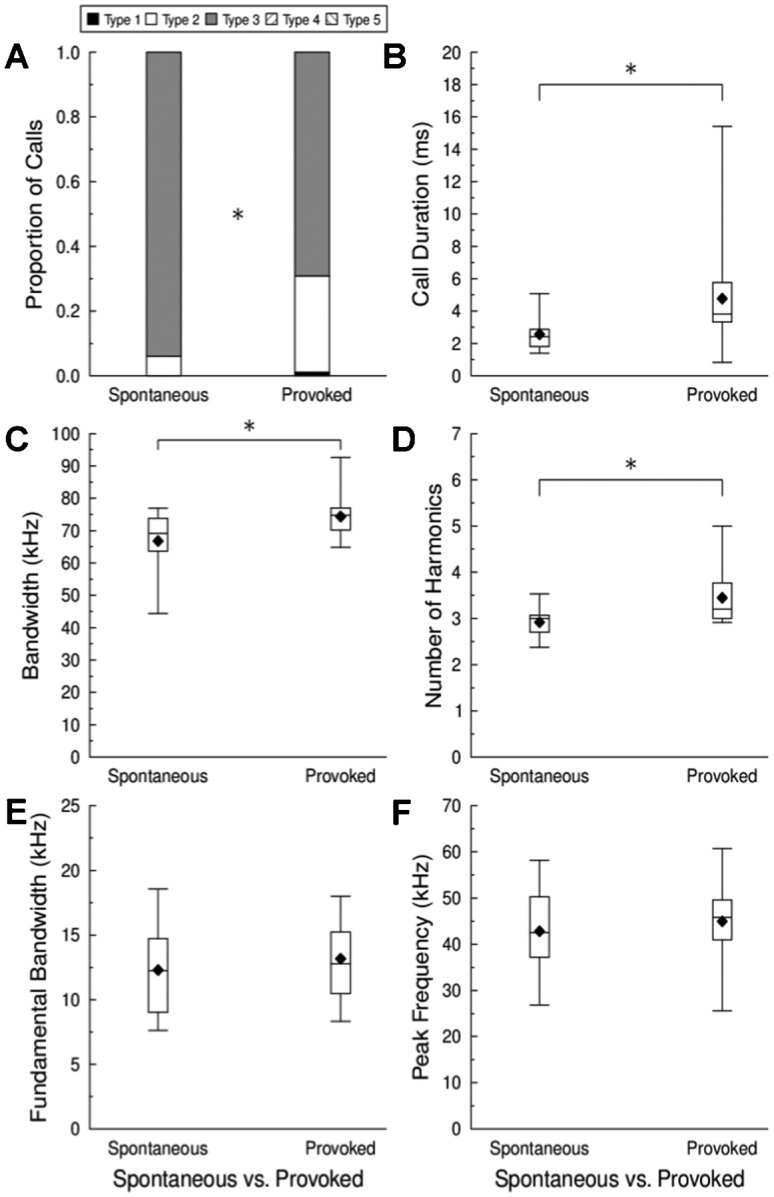
Change in call types and their acoustic parameters for spontaneous and provoked vocalizations of *E. fuscus* pups on PND 13 (n = 16). (A) Call type proportions for spontaneous and provoked vocalizations. Box and whisker plots comparing the (B) call duration, (C), signal bandwidth (D), number of harmonic elements (E), bandwidth of the fundamental acoustic element, and (F) peak spectral frequency of spontaneous and provoked vocalizations. The *line* within a box is the median, the *edges* are the 25^th^ and 75^th^ percentiles, the *whiskers* are the minimum and maximum values, and the *filled diamond* is the mean. * = p<0.05.

Our last experiment tested the hypothesis that human interactions would cause increased distress calling, leading to the prediction that provoked vocalizations would be more similar to younger-sounding vocalizations and thus more likely to promote maternal assistance/retrieval. We analyzed calls from 16 different pups on PND 13. Since we only analyzed data from PND 13 animals, each of the 16 pups provided one 30–60 second long spontaneous recording and one 30–60 second long provoked recording. The distribution of call types recorded for provoked *versus* spontaneous vocalizations was different (χ^2^ = 230.65, d.f. = 2, critical value = 13.82, p<0.001). Manually provoked pups emitted a larger proportion of Type I and Type II calls and fewer Type III calls compared to calls emitted spontaneously (Fig. 5A). Provoked calls were also longer in duration (Fig. 5B; t = −2.46, d.f. = 15, p = 0.027), larger in bandwidth (Fig. 5C; t = −3.08, d.f. = 15, p = 0.008) and contained more harmonic elements (Fig. 5D; t = −2.95, d.f. = 15, p = 0.010). This latter result was not surprising given that provoked calls were larger in bandwidth. There were no differences between spontaneous and provoked vocalizations in the bandwidth of the fundamental (Fig. 5E; t = −1.41, d.f. = 15, p = 0.180), peak spectral frequency (Fig. 5F; t = −0.89, d.f. = 15, p = 0.387), or any other acoustic parameter we measured (some data not shown).

## DISCUSSION

### Morphology and Behavior

The mass and forearm length of captive-born big brown bat pups increased at a rate of 0.40 g/day and 0.844 mm/day, respectively. There were no differences in the growth rates/trajectories of male and female pups (Fig. 1), a result consistent with previous observations (Kleiman, 1969; O'Farrell and Studier, 1973; Burnett and Kunz, 1982). Mass and forearm length were sexually dimorphic in adult bats; however, most collected females were pregnant and this would explain their higher mass. By PND 25, captive born male and female pups reached masses greater than wild-caught adults, likely because bats in captivity had *ad libitum* food, flew less, and experienced more stable temperatures. The majority of females gave birth to twins that often consisted of one female and one male pup, and there was no sex bias at birth which is consistent with other insectivorous bats (e.g. Brown, 1976).

Our behavioral observations on the timing of developmental milestones (e.g. eye opening, hair growth, attachment to the mother, and flight ability) were largely consistent with previous observations on *E. fuscus* development (Kleiman, 1969; Monroy et al., 2011). Gould (Gould, 1971) reported that the eyes of captive *E. fuscus* pups usually opened within a few hours after birth, but we found most pups opened their eyes between PND 2–3 (Fig. 1C). Compared to other bat species, *E. fuscus* pups opened their eyes at similar (Brown et al., 1983; Davis, 1969) or earlier ages (Kleiman, 1969; Orr, 1954; Habersetzer and Marimuthu, 1986; Jin et al., 2011) than other species. We observed *E. fuscus* pups starting to develop hairs by PND 4, while documented growth in other species varies between PND 2–8 (Kleiman, 1969). Other researchers have found pups not attached to their mother's nipple between PND 7–13 which is consistent with our observations (Fig. 1C) (Kleiman, 1969; Brown, 1976; McLean and Speakman, 1996).

Changes in the acoustic structure of pup calls may correlate with their new abilities of flight and independent aerial foraging, thus signalling the end of maternal dependence. As pups learn to echolocate and master the motors skills required for flight and catching nocturnal flying insects, this may stimulate a switch to emitting Type III calls (Fig. 3A). If so, we would expect pups to begin emitting biosonar-like calls just prior to or shortly after the onset of flight. Because *E. fuscus* pups began to emit Type III calls as early as PND 4/5, and flight attempts did not occur until approximately PND 21 (Fig. 1C), autonomous foraging cannot explain the switch from i-calls to biosonar calls. Alternative explanations for developmental vocal changes include the maturation of tissues (e.g. trachea, larynx, vocal cords, and muscles) involved in sound production (e.g. Kay and Pickvance, 1963; Matsumura, 1979), and/or extensive neuronal growth and circuit organization in brain regions used for hearing and phonation (e.g. Brown et al., 1978). A lactating female *E. fuscus* was captured from a day roost with an adult-sized pup attached to the teat estimated to be PND 36 (using the growth equations of Kunz, 1974; Brigham and Brigham, 1989), demonstrating that maternal dependence can last well past the age of first flight attempts (e.g. Kleiman 1969; Brown, 1976; Brown et al., 1983). *Myotis lucificus* and *M. thysanodes* pups made their earliest flight attempts at 19–20 and 16.5 days of age, respectively (Buchler, 1980; O'Farrell and Studier, 1973).

The growth equations constructed from healthy pup data up to PND 15 were as accurate in predicting pup ages as those that used data up to PND 25; however, the forearm length equation was a more accurate predictor than the mass equation, a finding consistent with a previous study (Burnett and Kunz, 1982). Accuracy in predicting pup ages may decrease over time because mass and forearm length variance increases with age (Fig. 1A,B). Moreover, mass can change over the course of a day (e.g. with variation in nutritional state and health), whereas forearm length is more stable. For researchers wishing to estimate ages of very young pups, we recommend using only the forearm length equation. Davis (Davis, 1969) and Burnett and Kunz (Burnett and Kunz, 1982) suggested that growth rates and equations, respectively, developed from bats in captivity with known and stable colony temperature and humidity parameters may not be applicable to wild bats, who experience more variable environmental conditions (Camaclang et al., 2006). Despite this concern, growth rates of our captive *E. fuscus* pups were similar to those reported from the field (mass: 0.3–0.47 g/day, forearm length: 0.8–1.4 mm/day (Burnett and Kunz, 1982)). Indeed, we found no evidence in the literature that *E. fuscus* captive growth was abnormal compared to the wild (see also Davis et al., 1968; Rajan and Marimuthu, 1999).

### Olfactory Discrimination Trials

Our olfactory discrimination trials were designed to determine if pups could identify and discriminate between the scents of their mother and the scent of another adult female—either from the same or different colony—or between their mother and the scent of another lactating female bat from the same colony (de Fanis and Jones 1996; Bloss et al., 2002). We were unable to find evidence that *E. fuscus* pups between PND 0–20 exhibited olfactory preferences for the scent of their mother (Table 1). These data suggest that *E. fuscus* pups have not formed scent preferences by PND 20; perhaps their developing olfactory nervous systems were still too immature and thus incapable of making individual odor discriminations that adult bats use to identify offspring. There is no evidence that *Tadarida brasiliensis mexicana* pups show a preference for muzzle or breast scent swabs of their mother *versus* another lactating female (Gustin and McCracken, 1987); however, other studies suggest that pups may recognize maternal odors and that olfactory preferences develop or change with age (Loughry and McCracken, 1991; de Fanis and Jones, 1996). In bats it has been suggested that reciprocal recognition occurs between mother and young mainly when the pups are older, whereas female identification predominates when the pups are younger (Hughes et al., 1989). In rats, maternal odors are not attractive to PND 1 pups but they are to PND 16 pups (for a review, see Leon, 1992). Because the onset and termination of pup attraction to maternal odors varies considerably across different rodent species (Leon et al., 1984), future studies must examine the temporal sequence of development of olfactory discrimination behaviour, olfactory receptors, and central olfactory circuits in bats.

### Vocal Development

The dominance of Type I i-calls early in development, when pups require the most maternal attention and assistance, was expected based on previous studies. Moreover, the spectral and temporal properties of *E. fuscus* i-calls (i.e. long duration, tonal signals with little to no FM) were consistent with many (but not all) previous reports on vocal development in bats (Brown, 1976; Gould 1971; Konstantinov, 1973; Matsumura 1979; Thomson et al., 1985; Koehler and Barclay, 1988; Moss, 1988; Kunz and Hood, 2000). Interestingly, Vater et al. (Vater et al., 2003) report that they were unable to record typical isolation calls from infant *Pteronotus parnelli*. We found that Type II calls were emitted at an intermediate phase of development when Type I call production was decreasing and Type III call production was increasing (Fig. 3A). Type II calls may be similar to the cruising pulses described by Gould (Gould, 1971).

Type III calls were spectrally and temporally similar to biosonar calls emitted by adult *E. fuscus* during foraging and navigation (Surlykke and Moss, 2000). The shift from emitting almost exclusively i-calls to biosonar-like calls has been reported by others studying big brown (Gould, 1971; Gould, 1975a; Gould, 1975b; Moss, 1988; Monroy et al., 2011) and other bats (Konstantinov, 1973; Gould, 1979; Matsumura 1979; Brown et al., 1983; Habersetzer and Marimuthu, 1986; Rübsamen, 1987; Sterbing, 2002; Liu et al., 2007; Vater et al., 2003). Gould (Gould, 1971) suggested that i-calls were precursors to biosonar signals emitted by adult bats. This idea is supported by some studies (e.g. de Fanis and Jones, 1995b; Moss et al., 1997; Sterbing, 2002; Wang et al., 2014); with mixed (Rübsamen, 1987) or opposing results from others (e.g. Brown et al., 1983; Jones et al., 1991; Vater at al., 2003; Knörnschild et al., 2007; Liu et al., 2007; Jin et al., 2011; Jin et al., 2012; Carter et al., 2014). Intermediate vocalizations with clear retrieval or echolocation functions have been reported by other researchers (Gould, 1971; Matsumura, 1979; Brown et al., 1983; Jones et al., 1991; Moss, 1988).

Despite differences in how calls were categorized, the ages where we saw *E. fuscus* pups shift from emitting one call type to another were largely consistent with those reported by Monroy et al. (Monroy et al., 2011). Type IV calls were similar to the single-arched FM social vocalizations of adult *E. fuscus* (Gadziola et al., 2012), and in pups may reflect an intermediate call type between i-calls and other social vocalizations. Type V calls were similar in duration to Type II and Type III calls, but spectrally were more similar to Type I calls and the quasi-constant frequency (QCF) social calls of adult *E. fuscus* (Gadziola et al., 2012). The function(s) of Type V calls remain unknown but because they were recorded early in development, they may serve as a maternal assistance/retrieval signal. Additional precursors to adult social calls may have been recorded if social interactions between mothers and pups had been permitted (e.g. Gould, 1971; Esser and Schmidt, 1988; Moss, 1998; Monroy et al., 2011). The Type II, IV and V calls reported in this study could also be intermediate calls and may reflect vocal learning, a lack of vocal-motor control, or possibly an undescribed form of pup communication. For example, the rate of contraction and relaxation of the cricothyroid muscles determines the frequency and duration of vocalizations in bats (Moss et al., 1997; Liu et al., 2007) and very young bats may not possess full control of their laryngeal musculature.

Our categorization of calls types was based initially on quantitative parameters and supplemented with qualitative measure, as opposed to other schemes relying primarily on descriptive/visual categories (e.g. Monroy et al., 2011). Quantitative classification should reduce the likelihood of misclassification based on subjective interpretations of call shape. Other researchers have described i-calls and echolocation calls using parameters such as duration and presence and direction of FM (Gould, 1971; Gould, 1975a; Gould, 1975b; Brown, 1976; Habersetzer and Marimuthu, 1986), as well as FM depth (shallow *versus* steep) and the presence/absence of FM on the initial/final portion of a call (Moss, 1988; Monroy et al., 2011).

As *E. fuscus* pups matured the duration of their calls shortened and the frequency and bandwidth decreased (Fig. 3B,D). This phenomenon has also been reported for the ontogeny of vocal development in several other species (Turner et al., 1972; Konstantinov, 1973; Brown et al., 1983; Brown, 1976; Scherrer and Wilkinson, 1993; Bohn et al., 2007; Knörnschild et al., 2007). Interestingly, i-calls emitted by some species increased in duration during development (Nelson, 1964; Vater et al., 2003; Liu et al., 2007), while in others i-calls first increased and then decreased in duration with age (Zhang et al., 2005; Jin et al., 2012). Still other species reported no overt changes for the acoustic parameters of i-calls (Schmidt et al., 1982; Van Parijs and Corkeron, 2002). *E. fuscus* i-call duration also increases with higher body temperatures (Camaclang et al., 2006). Isolation calls are loud and longer in duration than other pup or adult vocalizations. Together with their high repetition rate, this increases signal energy (Yost, 2007) making i-calls informative, conspicuous, and easier to localize (Marler, 1955). For pups attempting to attract their mothers' attention, longer duration signals may also be easier to identify and localize at a distance (e.g. Nelson, 1964). Echolocation signals are typically of very short duration which allows FM bats to emit more calls per unit of time while reducing the probability of and perceptual confusion caused by overlap between outgoing calls and returning echoes (Kalko and Schnitzler, 1989; Fenton et al., 2012).

The calls emitted by adult *E. fuscus* had larger (steeper) fundamental bandwidths and fewer harmonics than pup i-calls even though total signal bandwidth decreased only slightly during development (Figs 2, Fig. 3D,F). An increase in fundamental bandwidth with age was expected because the maximum fundamental frequency increased at a faster rate than minimum call frequency (Fig. 3G,H). Bats emitting FM echolocation calls likely use large fundamental bandwidths to increase the number of frequency (i.e. listening) channels and the resolution of auditory processing for localizing targets in three dimensions (Simmons and Stein, 1980; Zbinden, 1988; Bradbury and Vehnrencamp, 1998).

Minimum call frequency increased with age (Fig. 3H) while maximum call frequency remained relatively constant, which may reflect a developmental switch from emitting i-calls to echolocation calls (Gould, 1971; Konstantinov, 1973; Matsumura, 1979). Echolocation calls contain more energy at higher frequencies and young pups may be unable to produce sufficient tension on their vocal cords for such high frequency vocalizations (Colton, 1988). And because higher frequency sounds attenuate more rapidly in air than lower frequency sounds (Lawrence and Simmons, 1982; Marten and Marler, 1977; Marler, 1955), young pups may have been selected to use lower frequency sounds to attract their mothers.

Pup i-calls were multi-harmonic like many types of adult communication signals (Sterbing, 2002; Gadziola et al., 2012). In *E. fuscus* pups, the number of harmonics decreased while the peak spectral frequency of bat vocalizations increased with age, and such adjustments may reflect changes in vocal cord development. The natural/resonant frequency of vocal cord vibrations is determined by the stiffness, mass and tension of the vocal cords (Yost, 2007). Increased harmonic elements in i-calls may provide multiple reference points for interaural time and intensity difference comparisons for sound localization during mother-pup reunions (Marler, 1955). Alternatively, younger pups may not have the vocal control necessary to produce sounds with fewer harmonic elements, hence the harmonic richness in i-calls could reflect immature tracheal, laryngeal, vocal cord and/or muscular development. Griffin (Griffin, 1951), Konstantinov (Konstantinov, 1973); Brown (Brown, 1976); Vater et al. (Vater et al., 2003), and Liu et al. (Liu et al., 2007) all reported that the vocalizations of very young bats were rich in harmonics and audible to humans.

Adult *E. fuscus* calls consisted of downward FM sweeps with 2–3 harmonics. The presence of harmonics likely provides greater resolution for localizing prey and distinguishing insect targets from background clutter (Zbinden, 1988; Fenton 1997; Jones and Teeling 2006; Bates et al., 2011). FM chirps are also better than CF signals for range discrimination (Simmons and Stein, 1980; Jones and Teeling, 2006), which may also explain why adult bats continue to use multiple harmonic signals. When the number of harmonics increases, the total energy becomes more spread out across all frequencies. This effectively decreases signal energy per unit Hz and could influence the operating range of an echolocating bat. In situations requiring long distance communication, lower frequency calls with fewer harmonics would propagate through the atmosphere more efficiently (Lawrence and Simmons, 1982). Thus, an intermediate number of harmonics in echolocation calls might reflect a compromise between sophisticated localization and long-range communication.

### Prolonged Isolation Recordings

Although the use of call types changed with age—with PND 8 pups being more likely to produce Type II/III calls and PND 2 and PND 4 pups more likely to produce Type I/II calls (Fig. 4A)—we did not find evidence that older pups emitted younger-sounding calls during periods of maternal separation. Young *N. albiventris* pups were reported to emit short duration (<10 ms) CF and FM signals while crawling but longer duration CF and FM sounds when isolated or during other stressful situations (Brown et al., 1983).

With regard to temporal acoustic features, call repetition rate always decreased with increasing length of maternal separation, regardless of pup age (Fig. 4B). This result is consistent with findings from isolated *N. noctula* pups between PND 4–10 (Kleiman, 1969). In our study, *E. fuscus* pups on PND 2 emitted more calls and signalled for longer, with some individuals calling for up to 60 min post-separation. PND 4 pups ceased calling sooner and after 30 min none emitted calls, whereas none of the PND 8 emitted calls except after immediate separation (which may have been a consequence of human disturbance). Differences in calling rate at each developmental stage likely reflect differences in maturity, independence, and ability to go longer intervals without nursing (e.g. Esser and Schmidt, 1989). The decrease in call rate may also reflect an attempt by pups to conserve energy while separated from their mother. Alternatively, pups may become too tired or cold to continue signalling (Camaclang et al., 2006). Most pups on PND 2, and some on PND 4, increased their call durations with the length of maternal separation, perhaps suggesting that pups were attempting to increase signal energy and make their calls more similar to younger-sounding i-calls (Fig. 4C).

With regard to spectral features, if pups have been selected to produce younger-sounding vocalizations during maternal separation, then we would expect PND 2 and PND 4 pups to emit calls with more harmonics, higher maximum fundamental and peak spectral frequencies, and a lower minimum frequency with longer periods of isolation. This did not occur (Fig. 4D–F); very young pups may not have fine control over the spectral content of their vocalizations.

### Spontaneous vs. Provoked Vocalizations

PND 13 pups provoked by human observers changed their vocal repertoire to include fewer Type III (biosonar-like) and more Type I (isolation) and Type II (intermediate) calls compared to spontaneous vocal emissions recorded from the same pups (Fig. 5A). The observed changes were consistent with our prediction that provocation would induce older pups to emit younger-sounding calls, perhaps in an attempt to stimulate maternal assistance and/or retrieval. As hypothesized, provoked calls were longer in duration, had wider bandwidths, and an increased number of harmonics than spontaneous calls (Fig. 5B–D). The average bandwidths, durations and number of harmonics of the spontaneous PND 13 vocalizations were similar to the average bandwidths, durations, and number of harmonics recorded for all pups on PND 13; however, the bandwidths, durations and number of harmonics of provoked calls were more similar to those produced by PND 6, PND 7/8, and PND 9/10 pups, respectively (Fig. 3B,D,F), supporting the hypothesis that provoked pup vocalizations resemble those of younger pups. Gould (Gould, 1971) reported that *E. fuscus* pups increased their rate of calling during handling by humans, which is also consistent with our hypothesis. Additional research on anatomical changes to the vocal cords/tract and how this influences signal production in pups is needed to further our understanding on the development and complexity of the vocal repertoire of echolocating and non-echolocating mammals.

## MATERIALS AND METHODS

All procedures met the guidelines for the care and use of wild animals in research approved by the American Society of Mammalogists (Sikes et al., 2011), were approved by the Animal Research Ethics Board of McMaster University, and were in accordance with guidelines published by the Canadian Council on Animal Care.

### Animal Collection, Housing and Identification

Wild big brown bats (*Eptesicus fuscus*) captured between May 2006 and May 2011 were housed in a large husbandry facility at McMaster University where the colony temperature and lighting varied according to ambient conditions (Faure et al., 2009). Bats were given *ad libitum* access to food (mealworms, *Tenebrio molitor*) and water, and were permitted to fly within the colony. We studied 54 pregnant females that gave birth to 101 pups. Most females (47 of 54; 87%) gave birth to two pups (twins), with single births being less common (7 of 54 females; 13%; 2 female and 5 male pups). Twins typically consisted of one female and one male (24 of 47 adult female births) making visual identification easy; when working with same sex twins (female-female pups: 12 of 47 adult female births; male-male pups: 11 of 47 adult female births), temporary markings (e.g. felt pen on the skin) were used to identify pups (Mayberry, 2009).

### Morphology and Behavior

Pup growth and morphology were measured daily between PND 0–25, and when necessary every fifth day thereafter. Pups found attached to the teats of their mother were removed by placing a blunt probe into the pup's mouth and easing it open. Mass, forearm length and several developmental milestones were recorded, including: whether the pup was attached to the mother, if the pup's eyes were open, the presence/type of fur, and flight ability. Mass was measured with a Mettler PE3000 balance (resolution = 0.1 g), and forearm length with Manostat 15-100-100 vernier callipers (resolution = 0.01 mm). Flight ability was scored by placing the pup on a flat surface and either manually prodding it to fly or by holding the pup upside down by its feet and prodding it to fly. Five stages of flight ability were scored in a manner similar to Moss et al. (Moss et al., 1997): no attempt at flight (pup remained motionless), wing flapping (pup extends/flaps its wings without forward/upward motion), wing hopping (pup extends/flaps its wings with brief upward/forward motion), controlled falling (pup uses flapping to slow its descent but is unable to remain airborne), and true flight (pup performs powered flight and remains airborne). We also measured mass and forearm length data from wild caught *E. fuscus* adults for comparison with pup captive growth data (Mayberry, 2009).

#### Growth Equations and Age Estimation

Mass and forearm length data from pups of known ages were used to develop growth equations for estimating the age of unknown pups. The validity of the equations was tested with a random sub-sample of mass and forearm data not used for equation development. The predicted age of a pup was the average of the estimates from the mass and forearm length equations and compared to the pup's actual age in a new sub-sample of test pup data.

### Olfactory Discrimination Trials

Olfactory preferences of *E. fuscus* pups were tested at three developmental stages (PNDs 0–8, PNDs 9–14, and PNDs 15–20) with pups differing in their motor abilities. Discrimination trials took place in a plexiglass arena (30.5×23.5 cm; l×w×h) divided into 3 sections by ¼″ stainless steel mesh. For testing, pups were placed in the runway section of the arena (30.5×8 cm) that was adjacent to two odor test sections (15.25×15.5 cm). Wire mesh dividers permitted pups in the runway section to sample odors emanating from pieces of filter paper placed in both odor test sections, but prevented pups from physically entering these areas. Testing was conducted at room temperature (21–22°C) and in full lighting. Multiple identical arenas permitted us to test multiple pups daily. To minimize contamination with unwanted odors, the arenas with their dividers were cleaned in a commercial cage washer prior to their use. Additionally, the floor of each arena was swabbed with 100% ethanol, allowed to air dry, and lined with pieces of Whatman® filter. The filter paper was cut to the appropriate dimension for each arena section using scissors that had been wiped with 100% ethanol and handled wearing clean latex gloves.

Approximately 30–60 min prior to testing, pups were removed from a stainless steel wire mesh holding cage (28×20×20 cm) and isolated from their mother in separate holding cage in different room. This cage was placed under an infrared lamp to ensure pups remained warm and active prior to testing (Leon et al., 1984). Odors were collected by placing stimulus females on a clean piece of filter paper (14×8.5 cm) and covering them with clean glass jars. Stimulus females were handled by observers wearing clean latex gloves over leather gloves. Handling often caused stimulus females to urinate/defecate onto the filter paper thus impregnating it with natural odors (Brown, 1976; Leon 1992). Stimulus females were left on the filter paper for at least 15 min, after which they were returned to their holding cage and later reunited with their pups once they had completed testing. Filter papers were used within 2 hours of being impregnated with odors.

At the start of each trial, filter paper impregnated with a different test odor was placed on each side of the arena's odor testing section by an observer wearing clean latex gloves. The observer then donned a new pair of latex gloves before placing a test pup in the center area (neutral zone) of the runway, confined there with clean translucent dividers (28.5×0.5×15.5 cm; cleaned with 100% ethanol prior to use) to ensure an unbiased starting position. After 1 min, the translucent dividers were removed and the pup was permitted to freely crawl within the runway for 5 min. A perforated opaque divider (15.5×0.5×28.5 cm; cleaned with 100% ethanol prior to use) placed against the mesh ensured that visual cues from the odor test sections (e.g. staining on the filter paper) were not available to the pups. The arena was surrounded by taller white walls to ensure that distal room visual cues were not available to the pups. Testing took place in a quiet room isolated from other bats so that no auditory cures were available to the pups. A digital video camcorder (Panasonic Model PV-GS150) mounted above the arena recorded the pup's position in the runway during each trial. Runway position was scored as being on the left side, center area, or right side. Pups were considered to have crossed from one area to the other when more than fifty percent of the body had entered an area. Trials were quantified by the proportion of time that pups spent on the left or right side of the center area (i.e, time in proximity to each scent). Trials where the pup did not leave the center area were discarded.

We presented four types of filter paper as olfactory stimuli: (1) Blank: filter paper with no bat odor, (2) NF-SC: odor of non-reproductive adult female bat from the same colony as the mother, (3) NF-DC: odor of a non-reproductive adult female bat from a different colony as the mother, and (4) LF-SC: odor of a lactating adult female bat from the same colony as the mother. The test section assigned to each scent pairing was randomly determined and counter-balanced across the experiment.

### Vocal Recordings

We recorded the spontaneous vocalizations emitted by pups aged PND 0–25 with a CM16 condenser microphone (flat ±6 dB from 5–150 kHz; Avisoft Bioacoustics, Berlin, Germany) suspended 17 cm above an arena (30×36×16 cm) whose walls and floor were lined with 4 cm of sound attenuating foam to reduce echoes (Sonex® Classic; Pinta Acoustic, USA). Pups were removed from their mother and isolated in a separate room for recording (e.g. Scherrer and Wilkinson, 1993). Young, immobile pups were recorded directly from the arena center, whereas older and more mobile pups (which were more likely to crawl and hide in the foam) were sometimes recorded handheld (if necessary) with the microphone 17 cm away from the pup. Microphone output was digitized with an UltraSoundGate 116 (sampling rate 250 kHz, 16 bit amplitude resolution; Avisoft Bioacoustics) connected to a laptop computer running Avisoft Recorder software.

We also recorded echolocation calls of adult *E. fuscus* (n = 9) emerging from a maternity roost in the wild. Adult biosonar calls were recorded and analyzed with the same hardware and analysis software used for pup vocalizations.

### Sound Analysis and Call Types

Recordings were stored as .wav files and analyzed with Sound Analysis and Synthesis Laboratory Professional software (SASLab Pro, Avisoft Bioacoustics). The following acoustic parameters were measured from each call: duration, repetition rate, maximum frequency, minimum frequency, peak spectral frequency, total bandwidth, maximum and minimum frequency (and bandwidth) of the fundamental FM element, and number of harmonics. Duration (ms) was measured from the time domain display and defined as the end time minus the start time. Repetition rate (Hz) was calculated by counting the number of calls within a file and dividing by the recording time (30–60 s). The maximum, minimum, and peak spectral frequencies (Hz) of the call or the fundamental FM element were measured directly from the frequency domain (spectrogram) display. Because the fundamental FM element was the lowest spectral element of a call, the minimum frequency of the fundamental was also equal to the minimum call frequency (Fenton et al., 2011). The peak spectral frequency was defined as the frequency of maximum energy in the call. Bandwidth (Hz) was defined as the maximum minus the minimum frequency, and was calculated for both the entire call and the fundamental FM element. The number of harmonic elements was counted directly from the spectrogram display. Calls were classified into one of five types using both quantitative (duration) and qualitative parameters (presence and direction of FM).

### Prolonged Isolation Recordings

To determine if isolation from the mother altered the type, number, or acoustic structure of pup vocalizations, we recorded the vocalizations of PND 2, PND 4 and PND 8 pups during 120 min of maternal separation. Pups were separated from their mothers and placed in the recording arena (described above) and their spontaneous vocalizations (i.e. calls emitted without human interaction) were recorded at 15, 30, 45, 60, 90 and 120 min post-separation.

### Spontaneous vs. Provoked Recordings

To determine if human interaction caused pups to change their emitted call types or acoustic parameters, we compared spontaneous and provoked vocalizations of PND 13 pups (n = 16). Spontaneous vocalizations were recorded in the arena immediately after maternal separation and compared to a second, separate set of recordings collected from the same pups who were provoked (gentle prodding) to vocalize by a human observer.

### Statistical Analysis

Unless stated otherwise, all data are reported as the mean ± standard error (SE). One-sample t-tests (two-tailed) were used to determine if pups had olfactory preferences, testing against the null hypothesis of no preference and thus equal time spent with each scent (Bloss et al., 2002). For pups tested multiple times with the same scent pairing within the same age category, the average time spent in proximity to each scent was used in the analysis. Because not every pup emitted spontaneous vocalizations every day, we did not perform a daily repeated measures analysis on each acoustic parameter. Student's t-tests with sequential Bonferroni corrections (Rice, 1989) were used to compare the non-defining acoustic parameters of Type I, Type II and Type III pup calls. In the prolonged isolation study, a Chi-square test was used to compare the proportion of call types emitted by PND 2, PND 4 and PND 8 pups, and repeated measures ANOVAs were used to compare call parameters across 120 min of maternal separation for the pups that vocalized in each test session. Paired t-tests were used to compare the separation time when PND 2, PND 4 and PND 8 pups ceased calling (i.e. when the call repetition rate fell to 0 Hz), and to test for differences between the increments of separation that resulted in the highest and lowest values of each acoustic parameter. Chi-square tests were used to compare the proportions of call types and paired t-tests were used to test for differences in the parameters of spontaneous and provoked vocalizations emitted by PND 13 pups. All statistical tests employed an experiment-wise error rate of α≤0.05 (Zar, 1984).

### List of Abbreviations

dB, decibels; FM, frequency modulated; AM, amplitude modulated; 2-AFC, 2 alternative forced choice; i-call, isolation call; LF-SC lactating female same colony; NF-DC, non-reproductive female different colony; NF-SC, non-reproductive female same colony; PND, post-natal day; SD, standard deviation; SE, standard error; CF, constant frequency.

## References

[b1] Balcombe J. P. (1990). Vocal recognition of pups by mother Mexican free-tailed bats, Tadarida brasiliensis mexicana. Anim. Behav. 39, 960–966 10.1016/S0003-3472(05)80961-3

[b2] Balcombe J. P. McCracken G. F. (1992). Vocal recognition in mexican free-tailed bats: do pups recognize mothers? Anim. Behav. 43, 79–87 10.1016/S0003-3472(05)80073-9

[b3] Barbour R. W. Davis W. H. (1969). Bats of America Lexington, KY: The University of Kentucky Press.

[b4] Bates M. E. Simmons J. A. Zorikov T. V. (2011). Bats use echo harmonic structure to distinguish their targets from background clutter. Science 333, 627–630 10.1126/science.1202065 21798949

[b5] Bloss J. Acree T. E. Bloss J. M. Hood W. R. Kunz T. H. (2002). Potential use of chemical cues for colony-mate recognition in the big brown bat, Eptesicus fuscus. J. Chem. Ecol. 28, 819–834 10.1023/A:1015296928423 12035929

[b6] Bohn K. M. Wilkinson G. S. Moss C. F. (2007). Discrimination of infant isolation calls by female greater spear-nosed bats, Phyllostomus hastatus. Anim. Behav. 73, 423–432 10.1016/j.anbehav.2006.09.003 18311319 PMC2000849

[b7] Bradbury J. W. Vehnrencamp S. L. (1998). Principles of Animal Communication Sunderland, MA: Sinauer Associates.

[b8] Brigham R. M. Brigham A. C. (1989). Evidence for association between a mother bat and its young during and after foraging. Am. Midl. Nat. 121, 205–207 10.2307/2425674

[b9] Brown P. (1976). Vocal communication in the pallid bat, Antrozous pallidus. Z. Tierpsychol. 41, 34–54 10.1111/j.1439-0310.1976.tb00469.x 961121

[b10] Brown P. E. Grinnell A. D. Harrison J. B. (1978). The development of hearing in the pallid bat, Antrozous pallidus. J. Comp. Physiol. 126, 169–182 10.1007/BF00666371

[b11] Brown P. E. Brown T. W. Grinnell A. D. (1983). Echolocation, development, and vocal communication in the lesser bulldog bat, Noctilio albiventris. Behav. Ecol. Sociobiol. 13, 287–298 10.1007/BF00299676

[b12] Buchler E. R. (1980). The development of flight, foraging and echolocation in the little brown bat (Myotis lucifugus). Behav. Ecol. Sociobiol. 6, 211–218 10.1007/BF00569202

[b13] Burnett S. C. Kunz T. H. (1982). Growth rates and age estimation in Eptesicus fuscus and comparison with Myotis lucifugus. J. Mammal. 63, 33–41 10.2307/1380668

[b14] Camaclang A. E. Hollis L. Barclay R. M. R. (2006). Variation in body temperature and isolation calls of juvenile big brown bats, Eptesicus fuscus. Anim. Behav. 71, 657–662 10.1016/j.anbehav.2005.07.009

[b16] Carter R. T. Shaw J. B. Adams R. A. (2014). Ontogeny of vocalization in Jamaican fruit bats with implications for the evolution of echolocation. J. Zool. 293, 25–32 10.1111/jzo.12097

[b17] Christian J. J. (1956). The natural history of a summer aggregation of the big brown bat, Eptesicus fuscus. Am. Midl. Nat. 55, 66–95 10.2307/2422322

[b18] Colton R. H. (1988). Physiological mechanisms of vocal frequency control: the role of tension. J. Voice 2, 208–220 10.1016/S0892-1997(88)80079-1

[b19] Davis R. (1969). Growth and development of young pallid bats, Antrozous pallidus. J. Mammal. 50, 729–736 10.2307/1378249

[b20] Davis W. H. Barbour R. W. Hassell M. D. (1968). Colonial behavior of Eptesicus fuscus. J. Mammal. 49, 44–50 10.2307/1377726

[b21] de Fanis E. Jones G. (1995a). The role of odour in the discrimination of conspecifics by pipistrelle bats. Anim. Behav. 49, 835–839 10.1016/0003-3472(95)80215-0

[b22] de Fanis E. Jones G. (1995b). Post-natal growth, mother-infant interactions and development of vocalizations in the vespertilionid bat Plecotus auritus. J. Zool. 235, 85–97 10.1111/j.1469-7998.1995.tb05130.x

[b23] de Fanis E. Jones G. (1996). Allomaternal care and recognition between mothers and young in pipistrelle bats (Pipistrellus pipistrellus). J. Zool. 240, 781–787 10.1111/j.1469-7998.1996.tb05324.x

[b25] Esser K. H. Schmidt U. (1989). Mother-infant communication in the lesser spear-nosed bat Phyllostomus discolor (Chiroptera, Phyllostomidae) – evidence for acoustic learning. Ethology 82, 156–168 10.1111/j.1439-0310.1989.tb00496.x

[b26] Faure P. A. Re D. E. Clare E. L. (2009). Wound healing in the flight membranes of big brown bats. J. Mammal. 90, 1148–1156 10.1644/08-MAMM-A-332.1

[b27] Fenton M. B. (1985). Communication in the Chiroptera Bloomington, IN: Indiana University Press.

[b28] Fenton M. B. (1997). Science and the conservation of bats. J. Mammal. 78, 1–14 10.2307/1382633

[b29] Fenton M. B. Racey P. Rayner J. M. V. (1987). Recent Advances in the Study of Bats 119–134 Cambridge: Cambridge University Press.

[b30] Fenton M. B. Skowronski M. D. McGuire L. P. Faure P. A. (2011). Variation in the use of harmonics in the calls of laryngeally echolocating bats. Acta Chiropt. 13, 169–178 10.3161/150811011X578714

[b31] Fenton M. B. Faure P. A. Ratcliffe J. M. (2012). Evolution of high duty cycle echolocation in bats. J. Exp. Biol. 215, 2935–2944 10.1242/jeb.073171 22875762

[b32] Gadziola M. A. Grimsley J. M. S. Faure P. A. Wenstrup J. J. (2012). Social vocalizations of big brown bats vary with behavioral context. PLoS ONE 7, e44550 10.1371/journal.pone.0044550 22970247 PMC3436781

[b33] Gelfand D. L. McCracken G. F. (1986). Individual variation in the isolation calls of Mexican free-tailed bat pups (Tadarida brasiliensis mexicana). Anim. Behav. 34, 1078–1086 10.1016/S0003-3472(86)80167-1

[b34] Gould E. (1971). Studies of maternal-infant communication and development of vocalizations in the bats Myotis and Eptesicus. Commun. Behav. Biol. 5, 263–313.

[b35] Gould E. (1975a). Experimental studies of the ontogeny of ultrasonic vocalizations in bats. Dev. Psychobiol. 8, 333–346 10.1002/dev.420080407 1225700

[b36] Gould E. (1975b). Neonatal vocalizations in bats of eight genera. J. Mammal. 56, 15–29 10.2307/1379603 1113037

[b37] Gould E. (1979). Neonatal vocalizations of ten species of Malaysian bats (Megachiroptera and Microchiroptera). Am. Zool. 19, 481–491 10.1093/icb/19.2.481

[b38] Griffin D. R. (1951). Audible and ultrasonic sounds of bats. Experientia 7, 448–453 10.1007/BF02168686 14937008

[b39] Gunderson H. L. (1976). Mammalogy 307–332 New York, NY: McGraw-Hill Inc.

[b40] Gustin M. K. McCracken G. F. (1987). Scent recognition between females and pups in the bat Tadarida brasiliensis mexicana. Anim. Behav. 35, 13–19 10.1016/S0003-3472(87)80205-1

[b41] Habersetzer J. Marimuthu G. (1986). Ontogeny of sounds in the echolocating bat Hipposideros speoris. J. Comp. Physiol. A 158, 247–257 10.1007/BF01338568

[b42] Hood W. R. Bloss J. Kunz T. H. (2002). Intrinsic and extrinsic sources of variation in size at birth and rates of postnatal growth in the big brown bat Eptesicus fuscus (Chiroptera: Vespertilionidae). J. Zool. 258, 355–363 10.1017/S0952836902001504

[b43] Hughes P. M. Speakman J. R. Jones G. Racey P. A. (1989). Suckling behaviour in the pipistrelle bat (Pipistrellus pipistrellus). J. Zool. 219, 665–670 10.1111/j.1469-7998.1989.tb02607.x

[b45] Jin L. Lin A. Sun K. Liu Y. Feng J. (2011). Postnatal development of morphological features and vocalization in the pomona leaf-nosed bat Hipposideros pomona. Acta Theriol. (Warsz.) 56, 13–22 10.1007/s13364-010-0011-z

[b46] Jin L. Wang J. Zhang Z. Sun K. Kanwal J. S. Feng J. (2012). Postnatal development of morphological and vocal features in asian particolored bat, Vespertilio sinensis. Mamm. Biol. 77, 339–344 10.1016/j.mambio.2012.05.001

[b47] Jones G. Teeling E. C. (2006). The evolution of echolocation in bats. Trends Ecol. Evol. 21, 149–156 10.1016/j.tree.2006.01.001 16701491

[b48] Jones G. Hughes P. M. Rayner J. M. V. (1991). The development of vocalizations in Pipistrellus pipistrellus (Chiroptera: Vespertilionidae) during post-natal growth and the maintenance of individual vocal signatures. J. Zool. 225, 71–84 10.1111/j.1469-7998.1991.tb03802.x

[b49] Kalko E. K. V. Schnitzler H. U. (1989). The echolocation and hunting behavior of Daubenton's bat, Myotis daubentoni. Behav. Ecol. Sociobiol. 24, 225–238 10.1007/BF00295202

[b50] Kay L. Pickvance T. J. (1963). Ultrasonic emissions of the lesser horseshoe bat Rhinolophus hipposideros (Bech.). Proc. Zool. Soc. Lond. 141, 163–171 10.1111/j.1469-7998.1963.tb01607.x

[b51] Kleiman D. G. (1969). Maternal care, growth rate, and development in the noctule (Nyctalus noctula), pipistrelle (Pipistrellus pipistrellus), and serotine (Eptesicus serotinus) bats. J. Zool. 157, 187–211 10.1111/j.1469-7998.1969.tb01697.x

[b52] Knörnschild M. von Helversen O. (2008). Nonmutual vocal mother-pup recognition in the greater sac-winged bat. Anim. Behav. 76, 1001–1009 10.1016/j.anbehav.2008.05.018

[b53] Knörnschild M. von Helversen O. Mayer F. (2007). Twin siblings sound alike: isolation call variation in the noctule bat, Nyctalus noctula. Anim. Behav. 74, 1055–1063 10.1016/j.anbehav.2006.12.024

[b54] Koehler C. E. Barclay R. M. R. (1988). The potential for vocal signatures in the calls of young hoary bats (Lasiurus cinereus). Can. J. Zool. 66, 1982–1985 10.1139/z88-290

[b55] Konstantinov A. I. (1973). Development of echolocation in bats in postnatal ontogenesis. Period. Biol. 75, 13–19.

[b56] Kunz T. H. (1974). Reproduction, growth, and mortality of the vespertilionid bat, Eptesicus fuscus, in Kansas. J. Mammal. 55, 1–13 10.2307/1379252 4819591

[b58] Kunz T. H. Hood W. R. (2000). Parental care and postnatal growth in the Chiroptera. Reproductive Biology of Bats Crichton E G Krutzsch P H, ed415–468 New York, NY: Academic Press.

[b59] Kurta A. Baker R. H. (1990). Eptesicus fuscus. Mamm. Species 356, 1–10 10.2307/3504258

[b60] Lawrence B. D. Simmons J. A. (1982). Measurements of atmospheric attenuation at ultrasonic frequencies and the significance for echolocation by bats. J. Acoust. Soc. Am. 71, 585–590 10.1121/1.387529 7085967

[b61] Leon M. (1992). Neuroethology of olfactory preference development. J. Neurobiol. 23, 1557–1573 10.1002/neu.480231012 1487749

[b62] Leon M. Coopersmith R. Ulibarri C. Porter R. H. Powers J. B. (1984). Development of olfactory bulb organization in precocial and altricial rodents. Brain Res. 12, 45–53 10.1016/0165-3806(84)90175-5 6697256

[b63] Liu Y. Feng J. Jiang Y. L. Wu L. Sun K. P. (2007). Vocalization development of greater horseshoe bats, Rhinolophus ferrumequinum (Rhinolophidae, Chiroptera). Folia Zool. (Brno) 56, 126–136.

[b64] Loughry W. J. McCracken G. F. (1991). Factors influencing female-pup scent recognition in Mexican free-tailed bats. J. Mammal. 72, 624–626 10.2307/1382150

[b65] Marler P. (1955). Characteristics of some animal calls. Nature 176, 6–8 10.1038/176006a0

[b66] Marten K. Marler P. (1977). Sound transmission and its significance for animal vocalization. Behav. Ecol. Sociobiol. 2, 271–290 10.1007/BF00299740

[b67] Matsumura S. (1979). Mother-infant communication in a horseshoe bat (Rhinolophus ferrumequinum nippon): development of vocalization. J. Mammal. 60, 76–84 10.2307/1379760

[b68] Matsumura S. (1981). Mother-infant communication in a horseshow bat (Rhinolophus ferrumequinum nippon); vocal communication in three-week-old infants. J. Mammal. 62, 20–28 10.2307/1380474

[b69] Mayberry H. (2009). Development of Olfactory Discrimination in Big Brown Bats (Eptesicus fuscus) BASc thesis, McMaster University: Hamilton, ON, Canada.

[b70] McCracken G. F. (1984). Communal nursing in mexican free-tailed bat maternity colonies. Science 223, 1090–1091 10.1126/science.223.4640.1090 17830157

[b71] McCracken G. F. (1993). Locational memory and female-pup reunions in Mexican free-tailed bat maternity colonies. Anim. Behav. 45, 811–813 10.1006/anbe.1993.1094

[b72] McLean J. A. Speakman J. R. (1996). Suckling behaviour in the brown long-eared bat (Plecotus auritus). J. Zool. 239, 411–416 10.1111/j.1469-7998.1996.tb05464.x

[b73] Monroy J. A. Carter M. E. Miller K. E. Covey E. (2011). Development of echolocation and communication vocalizations in the big brown bat, Eptesicus fuscus. J. Comp. Physiol. A 197, 459–467 10.1007/s00359-010-0614-5 21327335

[b74] Moss C. (1988). Ontogeny of vocal signals in the big brown bat, Eptesicus fuscus. Animal Sonar: Processes and Performances (NATO ASI Series, Series A: Life Sciences), Vol. 156 Nachtigall P E Moore P W B, ed115–120 New York, NY: Plenum Press.

[b75] Moss C. F. Redish D. Gounden C. Kunz T. H. (1997). Ontogeny of vocal signals in the little brown bat, Myotis lucifugus. Anim. Behav. 54, 131–141 10.1006/anbe.1996.0410 9268443

[b76] Nelson J. E. (1964). Vocal communication in Australian flying foxes (Pteropodidae; Megachiroptera). Z. Tierpsychol. 21, 857–870 10.1111/j.1439-0310.1964.tb01224.x

[b77] O'Farrell M. J. Studier E. H. (1973). Reproduction, growth, and development in Myotis thysanodes and M. lucifugus (Chiroptera: Vespertilionidae). Ecology 54, 18–30 10.2307/1934371

[b78] Orr R. T. (1954). Natural history of the pallid bat, Antrozous pallidus (LeConte). Proc. Calif. Acad. Sci. 28, 165–246.

[b79] Porter F. L. (1979). Social behavior in the leaf-nosed bat, Carollia perspicillata. II. Social communication. Z. Tierpsychol. 50, 1–8 10.1111/j.1439-0310.1979.tb01012.x

[b80] Rajan K. E. Marimuthu G. (1999). Postnatal growth and age estimation in the Indian false vampire bat (Megaderma lyra). J. Zool. 248, 529–534 10.1111/j.1469-7998.1999.tb01052.x

[b81] Rasmuson T. M. Barclay R. M. R. (1992). Individual variation in the isolation calls of newborn big brown bats (Eptesicus fuscus): is variation genetic? Can. J. Zool. 70, 698–702 10.1139/z92-104

[b82] Rice W. R. (1989). Analyzing tables of statistical tests. Evolution 43, 223–225 10.2307/2409177 28568501

[b84] Rübsamen R. (1987). Ontogenesis of the echolocation system in the rufous horseshoe bat, Rhinolophus rouxi (audition and vocalization in early postnatal development). J. Comp. Physiol. A 161, 899–913 10.1007/BF00610231 3430416

[b85] Scherrer J. A. Wilkinson G. S. (1993). Evening bat isolation calls provide evidence for heritable signatures. Anim. Behav. 46, 847–860 10.1006/anbe.1993.1270

[b86] Schmidt U. Joermann G. Schmidt C. (1982). Struktur und variabilität der verlassenheitslaute juveniler vampirfledermäuse (Desmodus rotundus). Z. Säugetierkunde 47, 143–149.

[b87] Sikes R. S. Gannon W. L. **Animal Care and Use Committee of the American Society of Mammalogists**(2011). Guidelines of the American Society of Mammalogists for the use of wild mammals in research. J. Mammal. 92, 235–253 10.1644/10-MAMM-F-355.1 PMC5909806 29692469

[b88] Simmons J. A. Stein R. A. (1980). Acoustic imaging in bat sonar: echolocation signals and the evolution of echolocation. J. Comp. Physiol. 135, 61–84 10.1007/BF00660182

[b89] Sterbing S. J. (2002). Postnatal development of vocalizations and hearing in the phyllostoid bat, Carollia perspicillata. J. Mammal. 83, 516–525 10.1644/1545-1542(2002)083<0516:PDOVAH>2.0.CO;2 7928706

[b90] Surlykke A. Moss C. F. (2000). Echolocation behavior of big brown bats, Eptesicus fuscus, in the field and the laboratory. J. Acoust. Soc. Am. 108, 2419–2429 10.1121/1.1315295 11108382

[b91] Thomson C. E. Fenton M. B. Barclay R. M. R. (1985). The role of infant isolation calls in mother-infant reunions in the little brown bat, Myotis lucifugus (Chiroptera: Vespertilionidae). Can. J. Zool. 63, 1982–1988 10.1139/z85-290

[b92] Turner D. A. Shaughnessy A. Gould E. (1972). Individual recognition between mother and infant bats (Myotis). Animal Orientation and Navigation, SP-262 Galler S R Schmidt-Koenig K Jacobs G J Belleville R E, ed365–371 Washington, DC: National Aeronautics and Space Administration.

[b93] Van Parijs S. M. Corkeron P. J. (2002). Ontogeny of vocalisations in infant black flying foxes, Pteropus alecto. Behaviour 139, 1111–1124 10.1163/15685390260437281

[b94] Vater M. Kössl M. Foeller E. Coro F. Mora E. Russell I. J. (2003). Development of echolocation calls in the mustached bat, Pteronotus parnellii. J. Neurophysiol. 90, 2274–2290 10.1152/jn.00101.2003 14534267

[b95] Wang L. Lin A. Xiao Y. Jiang T. Feng J. (2014). Postnatal development in the big-footed bat, Myotis macrodactylus: wing morphology, echolocation calls, and flight. Acta Theriol. (Warsz.) 59, 435–441 10.1007/s13364-014-0182-0

[b96] Watkins L. C. Shump K. A. (1981). Behavior of the evening bat Nycticeius humeralis at a nursery roost. Am. Nat. 105, 258–268 10.2307/2424744

[b97] Wilkinson G. S. (1992). Communal nursing in the evening bat, Nycticeius humeralis. Behav. Ecol. Sociobiol. 31, 225–235 10.1007/BF00171677

[b98] Yost W. A. (2007). Fundamentals of Hearing: an Introduction, 5th edition San Diego, CA: Academic Press.

[b99] Zar J. H. (1984). Biostatistical Analysis, 2nd edition Englewood Cliffs, NJ: Prentice-Hall Inc.

[b100] Zbinden K. (1988). Harmonic structure of bat echolocation signals. Animal Sonar: Processes and Performance (NATO ASI Series, Series A: Life Sciences), Vol. 156 Nachtigall P E Moore P W B, ed581–587 New York, NY: Plenum Press.

[b101] Zhang L. Jones G. Parsons S. Liang B. Zhang S. (2005). Development of vocalizations in the flat-headed bats, Tylonycteris pachypus and T. robustula (Chiroptera: Vespertilionidae). Acta Chiropt. 7, 91–99 10.3161/1733-5329(2005)7[91:DOVITF]2.0.CO;2

